# Prevalence and Correlates of Depressive Symptoms among North Korean Defectors Living in South Korea for More than One Year

**DOI:** 10.4306/pi.2009.6.3.122

**Published:** 2009-08-03

**Authors:** Bong-Hee Jeon, Moon-Doo Kim, Seong-Chul Hong, Na-Ri Kim, Chang-In Lee, Young-Sook Kwak, Joon-Hyuk Park, Jaehwan Chung, Hanul Chong, Eun-Kyung Jwa, Min-Ho Bae, Sanghee Kim, Bora Yoo, Jun-Hwa Lee, Mi-Yeul Hyun, Mi-Jeong Yang, Duk-Soo Kim

**Affiliations:** 1 Department of Psychiatry and Institute of Medical Science, Jeju National University School of Medicine, Jeju, Korea.; 2 Department of Preventive Medicine, Jeju National University School of Medicine, Jeju, Korea.; 3 Department of Pediatrics, Masan Samsung Hospital, School of Medicine, Sungkyunkwan University, Masan, Korea.; 4 Department of Nursing Science, Jeju National University School of Medicine, Jeju, Korea.; 5 Jeju Mental Health Sanatorium, Jeju, Korea.; 6 Department of Chemistry, Jeju National University College of Natural Science, Jeju, Korea.

**Keywords:** Depressive symptoms, Correlates, North Korean defectors

## Abstract

**Objective:**

This study examined the prevalence and correlates of depressive symptoms in North Korean defectors who have been living in South Korea for more than one year.

**Methods:**

We used questionnaires developed by the authors to collect sociodemographic data in addition to the Center for Epidemiologic Studies Depression Scale (CES-D), the Psychosocial Well-being Index to measure stress, and a social support scale. A total of 367 subjects were included in this study.

**Results:**

The results showed that 30.5% of the men and 34.7% of the women reported depressive symptoms, and 33.1% of the men and 36.1% of the women exhibited signs of severe distress. Correlates of depressive symptoms were lack of occupation [odds ratio (OR)=2.198, 95% confidence interval (CI), 1.247-3.873], having escaped without family (OR=1.725, 95% CI, 1.006-2.959), and a poor subjective sense of health status (OR=3.111, 95% CI, 1.591-6.085).

**Conclusion:**

Continuing vocational training and career management, psychological support programs, and intensive physical health services are needed to improve the mental health of this population.

## Introduction

The number of North Korean defectors has been skyrocketing recently due to national economic and diplomatic crises, food shortages, weakened political power, diminishing loyalty to the Communist Party, and personal reasons.^1^ The number of North Korean defectors who have settled in South Korea has increased substantially since 1993, when eight North Korean defectors settled in South Korea. This number rose to 52 in 1994, 148 in 1999, 1,139 in 2002, 1,894 in 2004, 2,019 in 2006, 2,548 in 2007, and 2,809 in 2008. By the end of 2008, a total of 15,063 North Korean defectors had settled in South Korea.^2^

In response to the steady growth in the number of North Korean defectors settling in South Korea, government funding for settlement reached about one million dollars for 583 defectors in 2007, subsidies to employers for hiring reached about 2 million dollars in 2007, and other funds for supporting housing, education, and social security services have also increased continuously. Family violence, marital discord, divorce, runaways, adolescent defiance, and so on represent potentially major problems affecting the adaptation period.^3^ In addition, adolescents who are unable to assimilate to school life may experience alcohol addiction, interpersonal conflicts with other adolescents, and even involvement in crime.^4^ Problems arising during the period of adaptation can be exacerbated by unstable financial situations and social prejudice, possibly resulting in further increases in the crime rate.^5^

Psychological stressors related to this adaptation period include alienation from South Koreans, feelings of inferiority, loneliness, uncertainty about the future, concern about government retaliation against family members left in North Korea,^3^,^6^-^8^ and conflict between their North Korean government-controlled education about capitalism and their personal experiences with capitalism in South Korea.^8^

Countries other than South Korea have reported that long and unstable settlement processes^9^ and lowered social status affect the psychological stress experienced by refugees.^10^ Factors contributing to difficult adaptation have included family discord, discrimination, asylum procedures, low socioeconomic status, religion, vocational problems, and so on.^11^

These difficulties with the process of adaptation are clearly related to mental health.^9^,^12^-^14^ Chronic negative emotional experiences might harm the mental health of North Korean defectors^13^ and exacerbate underlying psychological problems, rendering adaptation difficult at best.^12^

Post-traumatic stress disorder (PTSD), anxiety disorders, and depressive disorders often affect recent escapees from North Korea.^15^ Studies of disasters have reported that depressive symptoms, anxieties, PTSD, psychosomatic syndromes,^16^-^20^ suicide attempts,^21^ and violence^22^ constitute major mental health problems among the affected populations.

However, the aforementioned research was primarily conducted immediately after escape or during the initial resettlement period. Research about the psychopathology of defectors who actually settle in South Korea remains lacking, and the literature is devoid of follow-up studies.

Although foreign studies have reported inconsistent results, agreement exists that the prevalence and severity of psychopathology tends to decrease with time.^23^ In particular, adolescents tended to recover rapidly recovery^24^ whereas elderly people tended to experience prolonged difficulties.^25^,^26^ In a study of North Korean defectors, Han^27^ reported that 29% among those living in Hanawon (a government-sponsored educational facility for the settlement of North Korean refugees during their initial phase in South Korea) experienced depressive symptoms.

Long-term follow-up studies of North Korean defectors are lacking, but Hong^28^ reported that the prevalence of subsyndromal PTSD had decreased from 31.8% to 5.3% after three years of resettlement, and the prevalence of full-blown PTSD decreased from 27.2% to 4.0% during this same period.

Until now, no studies about the correlates of depressive symptoms among North Korean defectors, especially among those who have settled South Korea, have been conducted. Thus, this study was conducted to investigate the prevalence and correlates of severe distress and depressive symptoms among people who have resided for at least one year in South Korea in order to provide baseline data with regard to North Korean defectors.

## Methods

### Sampling and period

The study was conducted from July 2006 to March 2007. The study population consisted of North Korean defectors over the age of 20 who had been living in Jeju-do, Busan metropolitan city, and Daegu metropolitan city in South Korea for more than one year. A total of 367 subjects were selected through centers supporting North Korean defectors. The sample consisted of 151 men and 216 women. Written informed consent was obtained from all participants.

### Measurements

#### Sociodemographic Data

Sociodemographic data on age, sex, marital status (in North Korea and South Korea), residential area (in North Korea and South Korea), education, occupation (in North Korea and South Korea), income (in North Korea and South Korea), religion (in North Korea and South Korea), party enrollment (in North Korea), military service (in North Korea), subjective sense of socioeconomic status (in North Korea and South Korea), time since escape from North Korea to South Korea, and family members participating in the escape were gathered via questionnaires developed by the authors.

#### Depressive Symptoms

The Korean version of the Center for Epidemiologic Studies Depression Scale (CES-D)^29^,^30^ was used to evaluate depression. This scale consists of 20 self-report questions designed to determine the existence and severity of depressive symptoms. We defined a CES-D score of 25 as the threshold for the purpose of estimating the prevalence of depression and a CES-D score of 21 as the threshold for the purpose of estimating the prevalence of depressive symptoms.^30^ Because the main goal of this study involved the early detection of depression, we used a score of 21 as the threshold for assessing the prevalence of depressive symptoms. Cronbach's α for this scale in this study was 0.892.

#### Level of Stress

We used the Psychosocial Well-being Index-Short Form (PWI)^31^ based on the General Health Questionnaire.^32^ This scale was developed for epidemiological studies rather than for clinical diagnosis. It consists of 18 questions scored on 4-point Likert scale from 0 to 3, with a total possible score of 54. Following the designers of this instrument, we defined positive well-being as scores under 8, moderate distress as 9 to 26, and severe distress as over 27. Cronbach's α for this scale in this study was 0.891.

#### Social Support Scale

The perceived level of social support was measured by the social support and social conflict items introduced by Abbey et al.;^33^ 11 questions scored on a 5-point Likert scale were included. The total possible score of this scale is 55, with higher score indicating greater social support. Cronbach's α for this study was 0.810.

#### Statistical Analysis

We estimated the prevalence of depressive symptoms among North Korean defectors according to sociodemographic characteristics, health status, family relationships, health habits, and degree of obesity. To identify the factors associated with depression, we first conducted logistic regression analysis for each potential factor after adjusting for age and sex. We then performed multiple logistic regression by including all the statistically significant factors in order to investigate their relationships with depressive symptoms. Odds ratios (OR) and corresponding 95% confidence intervals (CI) were used to measure the associations between depressive symptoms and the factors. Statistical Package for Social Science (SPSS) ver. 12.0 (SPSS Inc., Chicago, IL, USA), was used for all analyses; the level of significance was set at 0.05.

## Results

### Demographic characteristics of subjects

The total sample of 367 subjects consisted of 151 men and 216 women. The mean age of male subjects was 40.3 years (SD=14.0), that of female subjects was 40.6 years (SD=14.5), and that of the total sample was 40.4 years (SD=14.3). More than a majority of the sample (53.4%) resided in the City/district area of North Korea, 42.5% had been unmarried in North Korea, 24.4% had more than a college education, 63.6% had attended middle or high school, and 40.4% of male and 7.5% of female respondents had experienced military service, reflecting a statistically significant difference between the sexes in military experience. In terms of party enrollment, 34.7% of men and 8.5% of women had been members of the Communist Party, also reflecting a significant difference between the sexes. Nearly 60% (59.4%) of the subjects reported that their socioeconomic status in North Korea was low, and 55.6% of male and 56.9% of female participants had escaped from North Korea with family members.

In South Korea, 55.3% of the men and 58.8% of the women in the sample lived with spouses. Family incomes below 1.5 million won were reported by 8.8% of the sample, 9.4% of the male and 8.4% of the female respondents. Family incomes below 1 million won were reported by 57.1% of the male and 76.3% of the female participants. Of the total sample, 70.3% reported having occupations in South Korea; 71.5% of the men and 69.4% the women had occupations. Of the total sample, 75.6% reported identification with religion (Table 1).

### Health habits, stress, and depressive symptoms

In terms of consumption of alcoholic beverages, 40.7% of all subjects, 66.2% of the men and 22.8% of the women, report current drinking. Among this group, 26.1% of the total sample, 39.6% of the men and 15.6% of the women, obtained CAGE questionnaire scores greater than 2, indicating the possibility of alcohol dependence. More than one-third (34.9%) of the total sample, 33.1% of the men and 36.1% of the women, met criteria for severe distress. Nearly one-third (32.9%) of the total sample, 30.5% of the men and 34.7% of the women, reported depressive symptoms (CESD scores greater than 21). No significant differences between men and women emerged with regard to severe distress and depressive symptoms (Table 2).

### Prevalence and age- and sex-adjusted odds ratios for depressive symptoms by demographic variables

After controlling for sex and age, we calculated the ORs for depressive symptoms according to the values of the demographic variables. The prevalence of depressive symptoms according to residence before escape, marital status, education, military service experience, party enrollment, and socioeconomic status did differ significantly; however, those who identified with religion before escape were 3.579 times (95% CI, 1.569-8.162) more likely than were those without religion in North Korea to report depressive symptoms. Nearly 40% (39.4%) of persons who did not escape with family reported depressive symptoms, and this group was 1.680 times (95% CI, 1.080-2.612) more likely to have depressive symptoms than were those who escaped with family. The prevalence of depressive symptoms did not statistically differ according to marital status, place of residence in South Korea, time living in South Korea, and identification with religion. Those with family incomes below 1 million won were 6.092 times (95% CI, 1.802-20.593) more likely to report depressive symptoms than were those with family incomes greater than 1.5 million won. People without occupations were 2.289 times (95% CI, 1.386-3.780) more likely to report depressive symptoms than were those with occupations (Table 3).

### Prevalence and age- and sex-adjusted odds ratios for depressive symptoms by subjective health status, health-related behaviors, and stress

Those with a subjective sense that their health was poor were 3.460 times (95% CI, 1.885-6.349) more likely to experience depressive symptoms than were those with a subjective sense that they were in good health. Current smokers were 2.405 times (95% CI, 1.186-4.876) more likely than were nonsmokers to report depressive symptoms. People with CAGE scores greater that 2 were 2.454 times (95% CI, 1.407-4.281) more likely and those in the severe distress group were 9.355 times (95% CI 5.620-15.573) more likely to report depressive symptoms than were others (Table 4).

### Multiple logistic regression analysis of depressive symptoms by center for epidemiologic studies depression scale score and related variables

Multiple logistic regression analysis was conducted using the statistically significant variables in the simple regression analyses as well as those variables well known as risk factors for depressive symptoms as the independent variables; depressive symptoms were the dependent variable. Multiple logistic regression analysis showed that having no occupation (OR=2.198, 95% CI, 1.247-3.873), having escaped without family (OR=1.725, 95% CI, 1.006-2.959), and having a subjective sense that one's health was poor (OR=3.111, 95% CI, 1.591-6.085) were correlated with depressive symptoms (Table 5).

## Discussion

Subjects in this study were North Korean defectors who had been living in South Korea for more than one year after leaving Hanawon, a government-sponsored educational facility for settling North Korean refugees during their initial phase in South Korea. Male participants had lived in South Korea for a mean of 4.3 years (SD=1.5), female participants had lived in South Korea for a mean of 3.8 years (SD=1.7), and all participants had lived in South Korea for a mean of 4.0 years (SD=1.6). Of the 59.0% of the subjects living in South Korea for more than 4 years, 66.9% were male and 53.5% were female. The longest period of residence in South Korea was 13 years; the shortest period was one year.

Among North Korean defectors, depressive symptoms (CES-D scores greater than 21) were found in 30.5% of the men, 34.7% of the women, and 33.0% of the total sample, which is higher than previously reported results.^27^,^34^ Differences between this and previous studies might be attributable to differences in the characteristics of subjects, in that subjects in this study had lived in South Korea for more than one year, whereas most previous studies were conducted when defectors were in the initial education program in Hanawon. These results are also partially consistent with those of a previous study^35^ that found that psychopathology, including depression, tended to persist, but that the severity of PTSD tended to diminish as a function of time. The prevalence of depressive symptoms found by this study was higher than that found in the South Korean general population using the same CES-D scale: 23.1% among men and 27.4% among women.^36^ Thus, data show that North Korean defectors are more troubled than is the South Korean general population.

Although most studies report higher rates of depressive symptoms in women than in men, this study showed no significant differences in this regard. This result is consistent with previous research^37^,^38^ showing that North Korean male escapees experience less social support and tend to more depressed than do female North Korean escapees.

In this study, having no occupation, escaping without family, and having a subjective sense of one's health as poor were correlated with depressive symptoms.

Achievement of the personal goal of escaping from North Korea must constitute a main contributor to successful adaptation in South Korea. This study did not question subjects about their motives for escaping from North Korea. However, participants tended to be characterized by low socioeconomic status, urban residence, unmarried status, high school educations, and no history of army service; 7% of individuals leaving North Korea identified with religion and 43% left without family members. After settling in South Korea, 42.6% of respondents remained unmarried, 88.0% lived in urban areas, 68.7% had low monthly incomes (less than a million won per month), 72.5% were in a low socioeconomic group, and 29.7% were unemployed. The data pertaining to before and after escaping might not have shown subjective or objective improvements in socioeconomic status, but we could not confirm this possibility with statistical analyses. In other words, the socioeconomic status of defectors did not improve. The multiple logistic regression did not use family income as an independent variable, despite the high OR in the simple regression analysis, because 64.8% of the subjects earned less than 1 million won, and this variable was closely correlated with occupation. Continuous engagement in an occupation was thought to constitute the more important indicator of long-term income as well as a more central contributor to the emotional stability of the subjects, as compared to monthly income.^2^

Previous studies have shown that traumatic experiences^15^ in North Korea, accompanied by economic crises^1^ including food shortages, motivated respondents to escape from North Korea. North Korean escapees suffered a number of other psychological traumas during their long process of planning and executing escapes, and they seldom secured regular jobs after resettlement; more than 50% of the jobs involved performing simple labor^39^ and paid less than a million won per month. Research on the actual economic activities of North Korean defectors^39^ revealed that 67.8% held their jobs for less than one year, that 35.5% expressed dissatisfaction with their work places, a 10% increase over the comparable figure in 2007, and that 60.2% expressed discontent that their incomes did not meet their expectations. In addition, ambivalence^8^ about capitalism, deriving from conflict between the lessons learned in North Korea and the realities of capitalism experienced in South Korea, contributed to difficulties in adapting to South Korean society. Likewise, unemployment and reduced incomes constituted great barriers to successful adaptation to South Korea. On the other hand, regular jobs were associated with improvements in preexisting psychopathology and increased life satisfaction.^40^ Thus, efforts to provide stable occupations are necessary. Consistent with the results of previous studies,^34^ this study suggests that the financial difficulties of North Korean defectors represent the most important obstacles to successful adaptation to South Korean society.

Entry into South Korean society without family members represents another contributor to depressive symptoms. This finding is consistent with the results of a study focusing on foreign immigrants or refugees, but differs from the results of Han's research.^27^ This difference is due to differences in the characteristics of the subjects in Han's research,^27^ which included a small sample (n=64) of defectors who had stayed in Hanawon for only the initial period of settlement.

In addition, guilt about distance from families might lead to compensatory pressures for success,^14^ and the loneliness and alienation experienced by defectors might be amplified by the competition that represents a necessary evil of capitalism in South Korea. These factors might lead to the manifestation of depressive symptoms.

In general, a psychologically and physically strong person is considered to be highly likely to succeed in escaping from North Korea.^34^ Nevertheless, self-perceptions of poor health correlated with depressive symptoms. Subjects in this study were exposed to poor medical services while they were escaping from North Korea and were likely to confront health problems during the average of four years spent adapting to South Korea. Thus, participants had multiple concerns about their health status, and middle-aged participants faced many possible health problems. Indeed, 42.6% were living without spouses and were more likely to experience threats to their health due to bad health habits.

The principal limitation of our study is its cross-sectional design, which made it impossible to identify causal relationships between individual risk factors and depression. In addition, because this research did not use a nationwide sample, we cannot generalize our results to all North Korean defectors. The study was also limited by not including traumatic experiences as independent variables. However, this limitation is mitigated by previous research^28^ reporting that PTSD symptoms markedly decreased as a function of time.

In summary, mental health programs could provide early interventions for North Korean defectors experiencing such psychological problems as depressive symptoms that could otherwise detract from their quality of life and their adaptation to South Korean society.

A stable support system that provides vocational and technical education, not only in Hanawon but also as part of long-term follow-up to help defectors maintain jobs as they settle into South Korean society, is necessary to decrease the prevalence of depressive symptoms. Mental health programs are needed to address emotion problems related to guilt and depression for those who escape without families. In addition, programs to support physical health will improve not only physical health per se but also related symptoms of depression.

## Figures and Tables

**TABLE 1:**
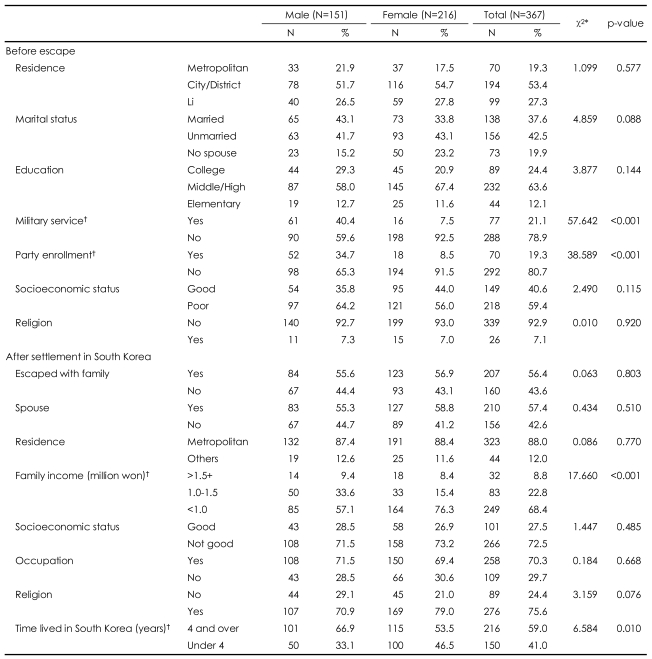
Demographic characteristics of subjects

^*^Test statistics from χ^2^-test, ^†^Statistically significant

**TABLE 2:**
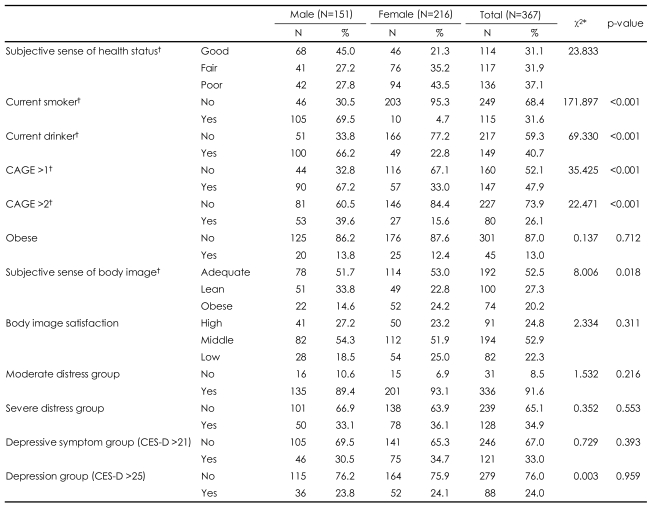
Health habits, stressors, and depressive symptoms

^*^Test statistics from χ^2^-test, ^†^Statistically significant. CES-D: center for epidemiologic studies depression scale

**TABLE 3:**
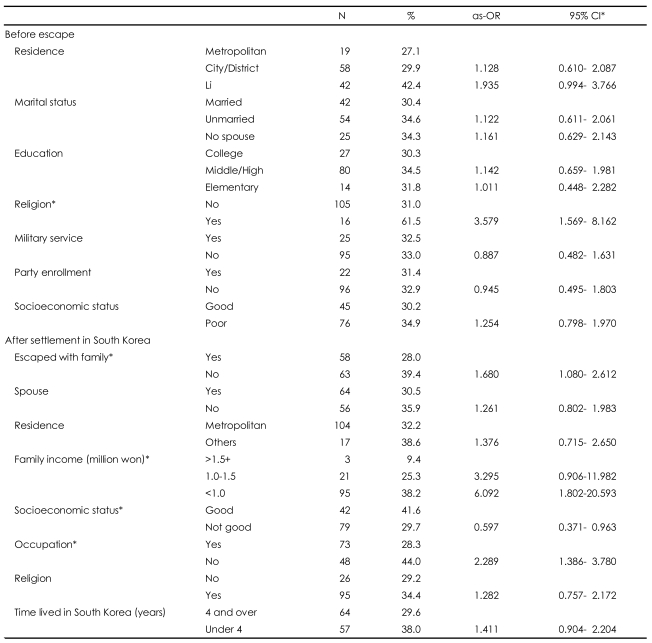
Prevalence and age- and sex-adjusted odds ratios (as-OR) for depressive symptoms by demographic variables

^*^Statistically significant. CI: confidence interval

**TABLE 4:**
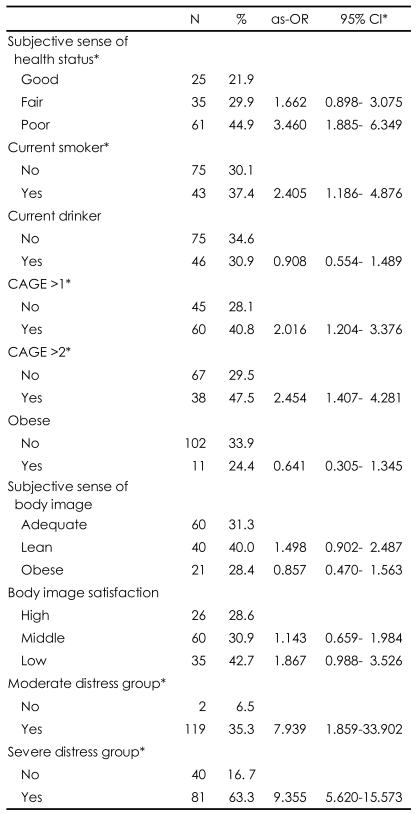
Prevalence and age- and sex-adjusted odds ratios (as-OR) for depressive symptoms by health status, health behaviors, and stressors

^*^Statistically significant. CI: confidence interval

**TABLE 5:**
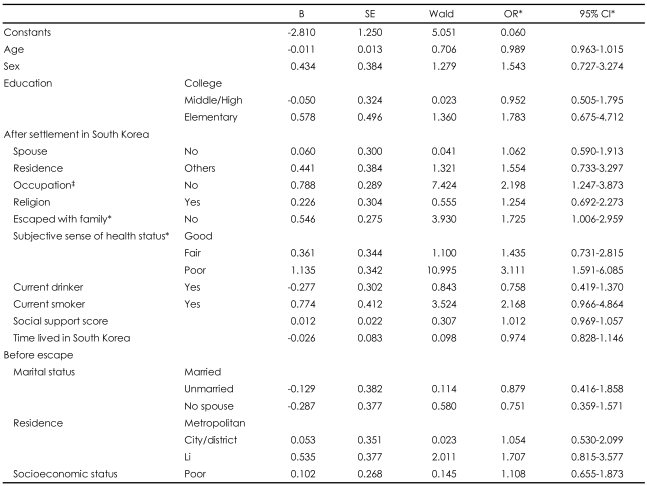
Multiple logistic regression analysis of depressive symptoms by CES-D scores and related variables

^*^Statistically significant. CES-D: center for epidemiologic studies depression scale, OR: odds ratio, CI: confidence interval
